# Black people in Ukraine: A content analysis of TikTok videos documenting discrimination against Black people attempting to flee at the onset of the 2022 Russo-Ukrainian war

**DOI:** 10.1016/j.dialog.2023.100161

**Published:** 2023-11-29

**Authors:** I.I. Vincent Jones, Sungwoo Kim, Haoyang Tang, Zhiheng Liu

**Affiliations:** a York College, City University of New York, Department of Health and Human Performance, Health Promotion Center, United States of America; b Teachers College, Columbia University, Department of Human Development, United States of America; c University of California, Santa Barbara, Department of Sociology, United States of America; d Columbia University, The Institute for Social and Economic Research and Policy, Graduate School of Arts and Sciences, United States of America

**Keywords:** Ukraine, War, Anti-blackness, Discrimination, Social media, Racism

## Abstract

This cross-sectional, descriptive study conducted on in June 2022 reviewed 100 TikTok videos using the hashtag #africansinukriane that depicted discrimination against Black people attempting to flee Ukraine at the onset of the war in February 2022. Two of the 16 themes were significant and present in over 50% of videos: raising awareness (67%) and racial discrimination (64%). Videos with elements of physical contact (*N* = 16, 76.2%), violence (*N* = 12, 75%), and dehumanization (*N* = 11, 68.8%) had higher shares than overall media shares. Less than 10% of the videos included dark humor (8%), sharing helpful resources (7%), and appreciation of countries that offered support (5%). Results indicate that videos that include raising awareness (*p* = .02), racial discrimination (*p* = .001), on-scene clips or war scenes (*p* = .007), physical contact (*p* = .006), and denied entry (*p* = .022). Their estimated differences in locations indicate that all of these themes were related to higher median shares of the videos. This study supports that TikTok is a place where marginalized groups can raise awareness about injustice and create counternarratives. This study exemplifies international anti-blackness with implications for health marketing and communication, human rights efforts, refugee health, and targeted mental health and policy support for those displaced by war.

## 1 Introduction

The Russo-Ukrainian War has been of global concern since 2014 following Ukraine's Revolution for Dignity [1]. This war escalated on February 24, 2022 when Vladimir Putin used the political status of Crimea as an impetus to declare war on Ukraine [2]. The invasion prompted at least 12 million people to flee, more than five million to neighboring nations [3]. From the outset of the Russo-Ukrainian War, media narratives implied that survival was linked to having white skin, and that escape proved more difficult for Black people [[4], [5], [6]]. Many of those stranded were international students with 16,000 African students residing in Ukraine as of 2022, comprising over 20% of international students [7,8].

Racism had life-threatening consequences for Black people in Ukriane. Racism is a social construct rooted in the belief of superiority based on skin color, placing Blacks as a subordinate group. Social media were critical in exposing the crisis faced by Black people, with videos depicting officials forcing them off trains, and even threatening to shoot African students at the boarder [9]. The racism observed during the war is not a new phenomenon in Ukraine. The literature documents racism in Ukraine against a backdrop of ethnic diversity, specifically against Russians, Romainis, Tartars, and Poles [[10], [11], [12], [13], [14]]. This discrimination extends to among the least represented groups such as African refugees, students, visitors, and citizens of African origin, who have lived under the unceasing menace of harassment and violence [15]. The war being waged between Russia and Ukraine has illuminated this undercurrent of racism [16].

There are several accounts of Black students being thwarted at border crossings, leaving them stranded for days in abject conditions [5,8,17,18]. Media depictions of these impediments sent global shockwaves. EuroNews illustrated how Black refugees were rejected and abused [19]. “We stayed [at the Poland-Ukraine border] from morning to night, in the cold, standing up, without food. We stayed four days without eating anything,” said Jean-Jacques Kabea, a Congolese pharmacy student who became a refugee during war [19].

We argue that anti-Blackness is the mechanism that renders Black people invisible, and prioritized white people's safe exit from Ukraine. “Anti-Blackness is defined as the beliefs, attitudes, actions, practices, behaviors and institutions that devalue, minimize, and marginalize the full participation of Black people visibly or perceived to be of African descent” [20]. Colorism can also collude to deal disadvantage along the lines of skin tone among melanated people at the boarder as well [21]. At the same time TikTok served as an effective means for them to raise awareness and garner international support that they otherwise might not have had.

Anti-Blackness has historically and continues to marginalize Black people [22]. This holds that the closer one is to whiteness both phenotypically and through social performance and capital, the more privileges they are ascribed. The history of wars is mostly Eurocentric, attempting to deemphasize the significance of other regions, especially Africa, in the anti-fascist conflict [15]. For example, the narratives of both World Wars highlight the pervasive hegemony of the West that underreports the contributions of Black people [23]. Chukwuokolo et al. (2022) posits how Africans are excluded from both the memorials and monuments that honor soldiers and their service in the two World Wars despite their invaluable contributions. This devaluation and erasure extends to the war in Ukraine where Black people refused to be erased and took to TikTok to make their voices heard.

### 1.1 Black TikTokers

TikTok is an app where people upload content between 15 and 60 s long. TikTok is especially popular among young people, and its features have created an environment where influencers, agencies, laypeople, and in this case those in crises, vie for attention within an attention economy [24]. Presently, TikTok, a community-sharing app, has 1.2 billion monthly active users and is expected to reach 1.8 billion by the end of 2022 [25]. The platform also boasts 100 million active users in the US, with 90% of them accessing the app daily [25]. Simultaneously while TikTok is banned in several countries (namely Pakistan, Indonesia and India) for political reasons and its potential to influence youth, the platform continues to find ways to circumvent regulations [26]. But just as much as racist sentiments circulate [27], TikTok has evolved into a hub for political discourse, activism, and social movements, especially among Black TikTokers who have recognized their cultural capital and influence [28]. They use the platform to address various social issues and combat instances of anti-Blackness, cultural appropriation, and exploitation [29,30], which has drawn the attention of non-TikTok users, highlighting the Black community's ability to leverage the platform to address racial politics [31]. For example, between June and July 2021, Black TikTokers boycotted the platform by withholding content, citing that white people were plagiarizing and profiting from their dances and challenges [28]. They disrupted the app and drew attention to their cause. While TikTok is globally regarded, and Black people have carved out their unique space within, this is the first content analysis to examine the experiences of Black people in wartime Ukraine through TikTok. Thus, the goal of this study was to describe the content of 100 TikTok videos that used the hashtags #africansinukriane. This study is significant as it is the first content analysis of any kind on this topic.

## 2 Methods

This cross-sectional, mixed-methods descriptive study was conducted in June 2022 on TikTok to examine the media related to Black people in wartime Ukraine. Basch and Basch's health-related social media content analysis methodology was used [32]. Their best practices were considered in formulating the research design, determining how many and which videos to view, deciding what to code, and ensuring the reliability of the same [32]. At the time of the study the most popular hashtags were #africansinukriane (9 M views) trailed by #africansinukraine (5.8 M views). Albeit misspelled, the first hashtag garnered the most traction among users, and was chosen for this study because it served as the most robust repository of media.

The data collection was carried out by a single person (HT). A query for the hashtag was run using TikTok's “discover” function and that rendered a list of videos in order of popularity at that time. Because the order is dynamic, all links to videos were collected in one session along with pertinent metadata to preserve the integrity of the order. This metadata included the date posted, and number of: views, overall likes, bookmarks, shares and comments. Videos with missing audio, in languages other than English without subtitles, or that were duplicated or miscategorized (i.e. were clearly unrelated to Blackness in Ukraine) were still cataloged, but marked for exclusion. HT continued until 100 usable videos were cataloged. 29 videos were excluded.

The following 16 themes were identified: raising awareness, human rights, call to action, media report, racial discrimination, appreciation of countries that offered support, expression of anger and sadness, live war scenes, violence, dehumanization, physical contact with law enforcement, students and young adults impacted, denial of entry (to transportation), uses of dark humor, somber tone, and access to helpful resources. All 100 videos were watched by a single reviewer (HT) who tagged videos based on the verbal content, written text, and the tone and lyrics of background music. The coding process involved a combination of inductive and deductive approaches, with initial themes identified (call to action, dehumanization, expression of anger or sadness, live war scenes, raising awareness, racial discrimination, violence), and additional themes emerging during the data collection process (Access to helpful resources, appreciation of countries that offered support, denial of entry, human rights, media reports, physical contact w/ law enforcement, somber tone, students and young adults impacted uses of dark humor). To ensure reliability and accuracy, the codebook guided the analysis of each video (see Table 1). A random sample of 25 videos from the spreadsheet were watched for the same content by a second reviewer (ZL). The reviewers agreed on all datapoints collected resulting in a reliability score of *κ* *=* *1.* Descriptive statistics were recorded in Microsoft Excel and analyzed in R. The York College (CUNY) Institutional Review Board determined that a review was unnecessary because the study did not involve human subjects.

**Table 1:** Codebook Definitions for Videos Tagged #Africansinukriane.

1. Access to Helpful Resources	Creators provide information for civilians to learn where to access resources, aid, and support for those in Ukraine, and for those trying to leave.
2. Appreciation of Countries that Offered Support	Show of appreciation, thanks, and or gratitude for specific countries or organizations specific to certain countries that have provided aid, resources, support, or assistance for the war.
3. Call to Action	Content that specifically encourages a call to action in the form of signing a petition, donating money, participating in a campaign, or some form of activism.
4. Dehumanization	Instances of unfair or unequal treatment, prejudice, or bias directed toward people of color in the videos; degrading and foul language.
5. Denial of Entry	Words, actions, or stories of people showing of Black people being denied entry to transportation leaving Ukraine.
6. Expression of Anger or Sadness	Depiction of anger or sadness without somber music or melody
7. Human Rights	Demonstration of explicit or implicit violation of fundamental rights such as dignity, equality, and respect faced by Black people in Ukraine.
8. Live War Scenes	Footage showing active war scenes or previously recorded content where shooting, battles, conflicts involving violence between Russian combatants and Ukrainian civilians, law enforcement–or the destruction of infrastructure by military operations.
9. Media Report	Information showing reports from any form of news or media outlets/sources
10. Physical Contact with Law Enforcement	Show of force by law enforcement when dealing with Black people in Ukraine.
11. Raising Awareness	Mention of the need to promote awareness to a topic, issue, cause with an emphasis on inspiring others to engage in action or to support change.
12. Racial Discrimination	Content that is specific to discrimination or highlights synonymous terms, i.e., prejudice, bias, or inequality.
13. Somber Tone	Music or tone that has a slow tempo and shows a sad or reflective moment or has words that indicate loss or introspection.
14. Students and Young Adults Impacted	Content specific to the experiences of people who were identified as students (by others or self-identified) or young adults through visual identification–along with information on consequence and outcomes related to war experiences.
15. Uses of Dark Humor	Sarcasm or making light of the situation faced by Black People in Ukraine and other matters that would otherwise be inappropriate to joke about.
16. Violence (physical)	Use of force with the intent to inflict harm, damage, or injury to another person such as physical altercations, armed conflicts, domestic abuse, or acts of aggression.

## 3 Results

A total of 100 videos were included in the analysis, which generated 15,100,838 views, 444,199,305 likes, 89,843 comments, 1,283,425 bookmarks, and 142,996 shares. Due to the extreme skewness of the variables, medians were computed and selected as the central value (Refer to Appendix A for histograms of each variable. The median (standard deviation) of each variable is as follows: 29,000 (450,368.28), 287,900 (13,527,251.74), 320.5 (1629.24), 3277 (29,298.20), and 279.5 (3854.30).

Table 2 shows 16 categories of various characteristics that were coded from the videos. The table includes the number of videos (N), total number of views, percent of total views from grand total views (% Total), and percentage of videos that also had higher than median views (% Over Median) for each category. These values were also computed for likes, comments, bookmarks, and shares. (See Table 3.)

**Table 2:** Observed content, views, like, comments, hearts, and shares of 100 TikTok videos on #AfricansinUkraine.

Observed Content		Views			Likes			Comments			Bookmarks			Shares		
	N	Total	% Total	% Med	Total	% Total	% Med	Total	% Total	% Med	Total	% Total	% Med	Total	% Total	% Med
Raising Awareness	67	10,390,570	68.8%	52.2%	260,483,526	58.6%	55.2%	58,114	64.7%	52.2%	951,002	74.1%	52.2%	108,080	75.6%	55.2%
Human Rights	26	3,681,923	24.4%	65.4%	93,391,888	21.0%	80.8%	30,831	34.3%	65.4%	414,366	32.3%	65.4%	39,909	27.9%	61.5%
Call to Action	20	1,855,360	12.3%	45.0%	53,110,388	12.0%	55.0%	19,195	21.4%	45.0%	373,075	29.1%	55.0%	65,926	46.1%	60.0%
Media Report	24	4,355,700	28.8%	62.5%	182,911,827	41.2%	50.0%	35,287	39.3%	66.7%	369,918	28.8%	54.2%	35,027	24.5%	62.5%
Racial Discrimination	64	9,253,323	61.3%	59.4%	298,981,206	67.3%	53.1%	70,972	79.0%	57.8%	789,367	61.5%	57.8%	80,185	56.1%	60.9%
Appreciation of Countries which Offered Support	5	208,254	1.4%	40.0%	1,864,600	0.4%	20.0%	1551	1.7%	40.0%	16,759	1.3%	40.0%	892	0.6%	20.0%
Expression of Anger and Sadness	18	2,083,024	13.8%	61.1%	82,161,700	18.5%	44.4%	22,516	25.1%	55.6%	301,449	23.5%	72.2%	31,964	22.4%	61.1%
Live War Scene	42	4,778,747	31.6%	64.3%	128,357,996	28.9%	42.9%	34,832	38.8%	59.5%	531,595	41.4%	52.4%	85,992	60.1%	64.3%
Violence	16	1,905,600	12.6%	81.3%	90,059,300	20.3%	50.0%	11,890	13.2%	62.5%	147,137	11.5%	56.3%	19,751	13.8%	75.0%
Dehumanization	16	1,802,924	11.9%	56.3%	39,904,400	9.0%	50.0%	16,862	18.8%	75.0%	311,474	24.3%	62.5%	50,431	35.3%	68.8%
Physical Contact with Law Enforcement	21	2,696,500	17.9%	76.2%	83,358,100	18.8%	47.6%	17,229	19.2%	61.9%	182,474	14.2%	57.1%	27,401	19.2%	76.2%
Students and Young Adults Impacted	29	3,808,323	25.2%	48.3%	103,101,715	23.2%	58.6%	34,050	37.9%	55.2%	607,980	47.4%	51.7%	78,602	55.0%	58.6%
Denied Entry	41	6,591,524	43.7%	61.0%	283,594,127	63.8%	48.8%	49,695	55.3%	63.4%	595,626	46.4%	56.1%	55,579	38.9%	63.4%
Uses of Dark Humor	8	4,368,800	28.9%	37.5%	11,417,380	2.6%	37.5%	4142	4.6%	37.5%	147,818	11.5%	37.5%	4834	3.4%	25.0%
Somber Tone	21	2,579,723	17.1%	47.6%	12,432,896	2.8%	38.1%	20,462	22.8%	42.9%	392,232	30.6%	38.1%	57,848	40.5%	33.3%
Access to Helpful Resources Sharing	7	106,190	0.7%	0.0%	85,182,200	19.2%	57.1%	891	1.0%	14.3%	20,768	1.6%	42.9%	6060	4.2%	28.6%

**Table 3:** Mann Whitney Tests: Characteristics that produced significant differences in the number of shares.

	N	W	*p*-value	Estimated Difference in Location
Raising Awareness	67	790	0.021*	−145
Human Rights	26	747	0.092	−161.46
Call to Action	20	682	0.311	−149.87
Media Report	24	749	0.19	−94.3
Racial Discrimination	64	694.5	0.001*	−210
Expression of Anger and Sadness	18	605.5	0.236	−177.02
Live War Scene	42	833.5	0.007*	−212
Violence	16	496	0.098	−210
Dehumanization	16	475	0.065	−245.65
Physical Contact	21	505.5	0.006*	−268
Students Impacted	29	931.5	0.459	−44
Denied Entry	41	882.5	0.022*	−177
Somber Tone	21	936.5	0.367	69

Two characteristics were found to be present in over 50% of the videos, which were raising awareness (67%) and racial discrimination (64%). Less than 10% of the videos included dark humor (8%), sharing helpful resources (7%), and appreciation of countries that offered support (5%).

Multiple characteristics had a higher tendency to be present in relatively highly shared videos. Videos with elements of physical contact involving law enforcement (*N* = 16, 76.2%), violence (*N* = 12, 75%), and dehumanization (*N* = 11, 68.8%) had higher shares than overall media shares.

Due to the skewness of the data and significant violation of Shapiro-Wilk normality test (*p* < .001), Mann-Whitney *U* tests were conducted instead of independent samples *t*-test to identify characteristics that produced significant differences in the number of shares. Results indicate that videos that included raising awareness (*p* = .02), racial discrimination (*p* = .001), live war scenes (*p* = .007), physical contact involving law enforcement (*p* = .006), and denied entry (*p* = .022). Their estimated differences in locations indicate that all of these videos were related to higher median shares of the videos.

## 4 Discussion

This study highlights challenges within a sample of TikTok videos about being Black in Ukraine at the onset of the war. Two of the themes were present in over 50% of the videos: 1) raising awareness (67%) and racial discrimination (64%).

The theme of raising awareness is aligned with one of the purposes of social media: to empower laypeople to share experiences with immediacy and reach that could otherwise go unheard [[33], [34], [35]]. Given that the students were trapped, social media was a reliable way to bring attention to their emergency [9], thus the theme of raising awareness is pervasive.

Additionally, it's inevitable that many videos focused on raising awareness also include content about racial discrimination because they encompassed topics such as racism, hate crimes, and racial bias, consistent with rhetoric observed within organizations like the United Nations [36], the Department of Justice [37] Brookings Institute [38] and higher education institutions [39,40] regarding inequality.

This study also highlights the significant number of shared videos related to the hashtag #AfricansInUkriane. While content related to raising awareness and racial discrimination garnered high levels of engagement through shares, videos featuring themes like ‘physical contact involving law enforcement,’ ‘war scenes,’ and ‘denied entry’ also received significant shares. All these themes contribute to the broader discussion of African people in Ukraine, a central focus of the hashtag. Thus, there was often overlap between the themes. However, the question remains: why were these themes more prevalent? One possibility is that the stark realities of war and its associated imagery captivated people. At the onset of the war, people were curious about what was happening on the ground, and the media from the aggrieved Black people was arresting. Viewers were captivated by law enforcement's misuse of force to oppress Black people.

TikTok emerged as a major platform for witnessing the crisis faced by people in Ukraine, with videos tagged #UkraineWar accumulating over 600 million views [41]. This fascination has not only drawn the attention of historians but also health professionals [42]. Through social media, people are galvanized to educate themselves, inform others, and bear witness to atrocities [42].However, it is important to note that while this type of content generates high share engagement, excessive exposure to such events can have deleterious mental health consequences, an area that health professionals have found necessary to investigate [42].

### 4.1 Limitations

While this cross-sectional study is useful in providing preliminary evidence on content that has resulted in significant engagement, it reflects one historical timepoint, and is limited as video content and viewership change over time. Additionally, the data were collected by one researcher in one sitting, which could potentially introduce researcher fatigue. The presence of overlapping themes made it challenging to clearly delineate differences between some themes. We attempted to mitigate the said limitations with a codebook and an interrater reliability check. Nevertheless, this study makes a meaningful contribution to the literature as it relates to social media as an effective tool to examine anti-Blackness and other forms of oppression.

### 4.2 Recommendations

Future studies may consider collecting viewer comments to examine the subsequent interaction and dialogues that emerge. They could also focus on the role of social media in holding law enforcement and even nation states accountable for their mistreatment of others. Given that TikTok users may be different than those on other platforms, this study should be replicated on other social media apps.

## 5 Conclusion

This is another example of social media proving an effective tool for bringing attention to those in crisis. Tiktok is a conduit for political engagement, online activism, and a means to promote public health iniquities for underrepresented groups. This scholarship is especially important because it contributes a Black counter narrative that could otherwise be lost, as research suggests that their contributions are often overlooked in wartime. Social media has promoted prolific political participation and perpetuated social change around the world. As media affirm reality, the online calls to action support the building of offline communities and norm formation. The results from this study, the first TikTok content analysis on this topic, emphasize the need for mental health and policy resources for those directly and vicariously affected by the ongoing war in Ukraine. Additionally, this research highlights another concerning issue: people's inclination toward content featuring negative war imagery, abuse and injustice, much of which can have immediate effects on their mental health.

## Authors' contributions

VJ conceptualized the study. HT collected the data and SK conducted the data analysis. All authors contributed to the manuscript production.

## Ethics approval

This research study did not involve human subjects. Thus, the York College CUNY Institutional Review Board (IRB) determined that it did not require review.

## Statement

Declaration of generative AI in scientific writing.

Statement: No artificial intelligence was used to prepare this work.

## Declaration of Competing Interest

The authors declare the following financial interests/personal relationships which may be considered as potential competing interests:

DR VINCENT JONES II reports article publishing charges were provided by The Andrew W. Mellon Foundation and The City University of New York Black Race and Ethnic Studies Award (7W209-06 01).

## Appendix A Supplementary data

Histograms Image 1
