# Supplementary figures and images for: Black people in Ukraine: A content analysis of TikTok videos documenting discrimination against Black people attempting to flee at the onset of the 2022 Russo-Ukrainian war
Source: Dialogues Health. 2023 Nov 29;4:100161. doi: 10.1016/j.dialog.2023.100161 (PMC10953848; doi:10.1016/j.dialog.2023.100161)

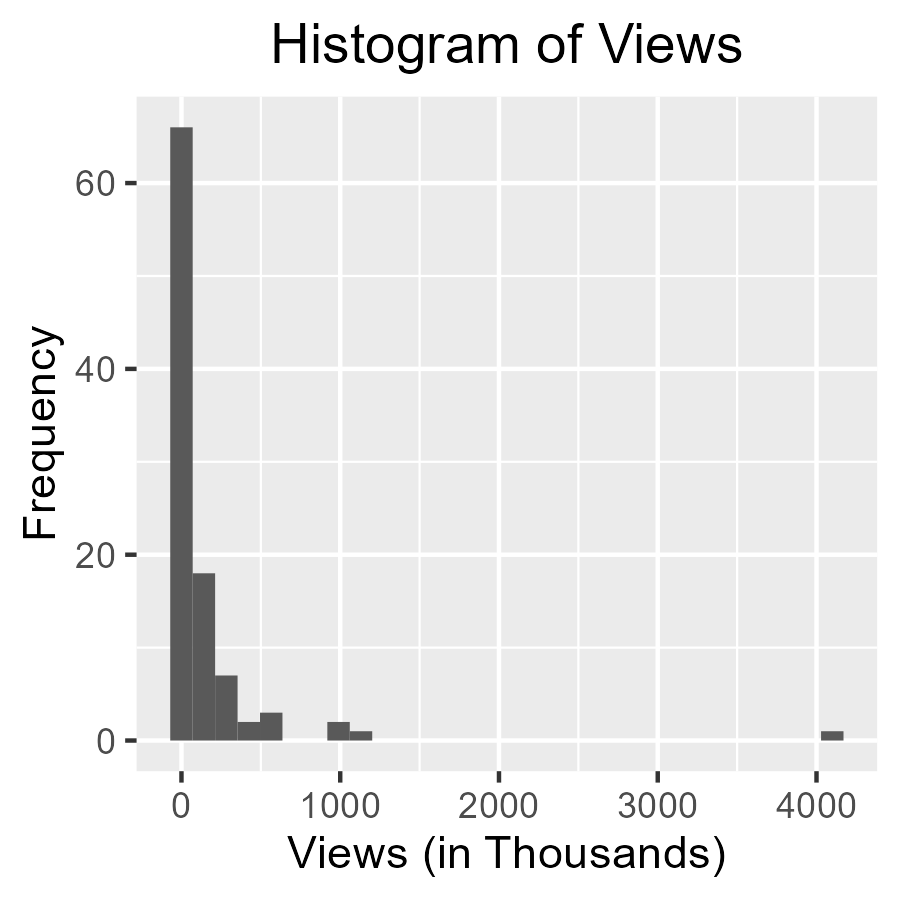

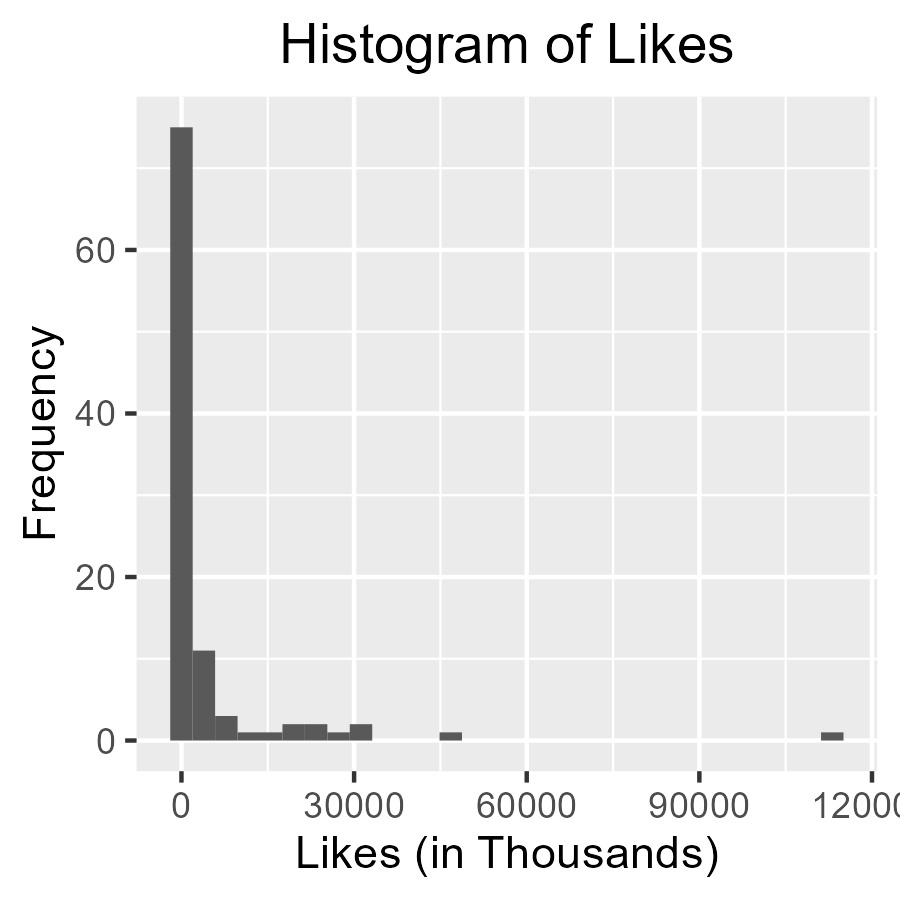

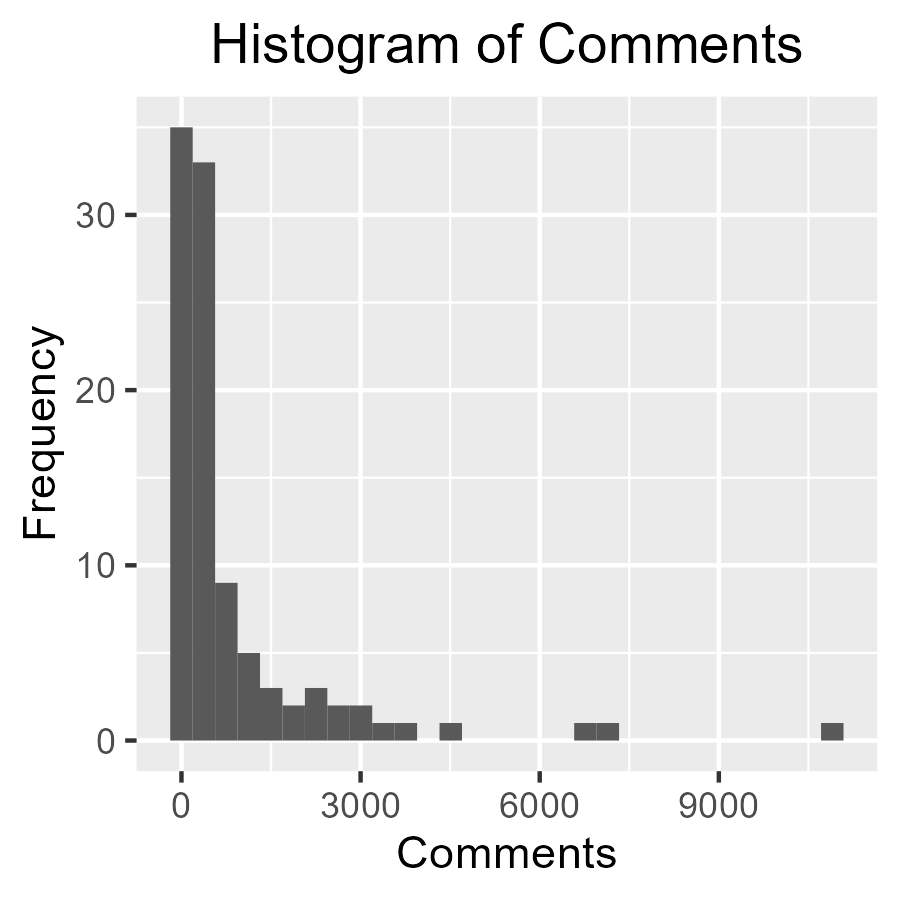

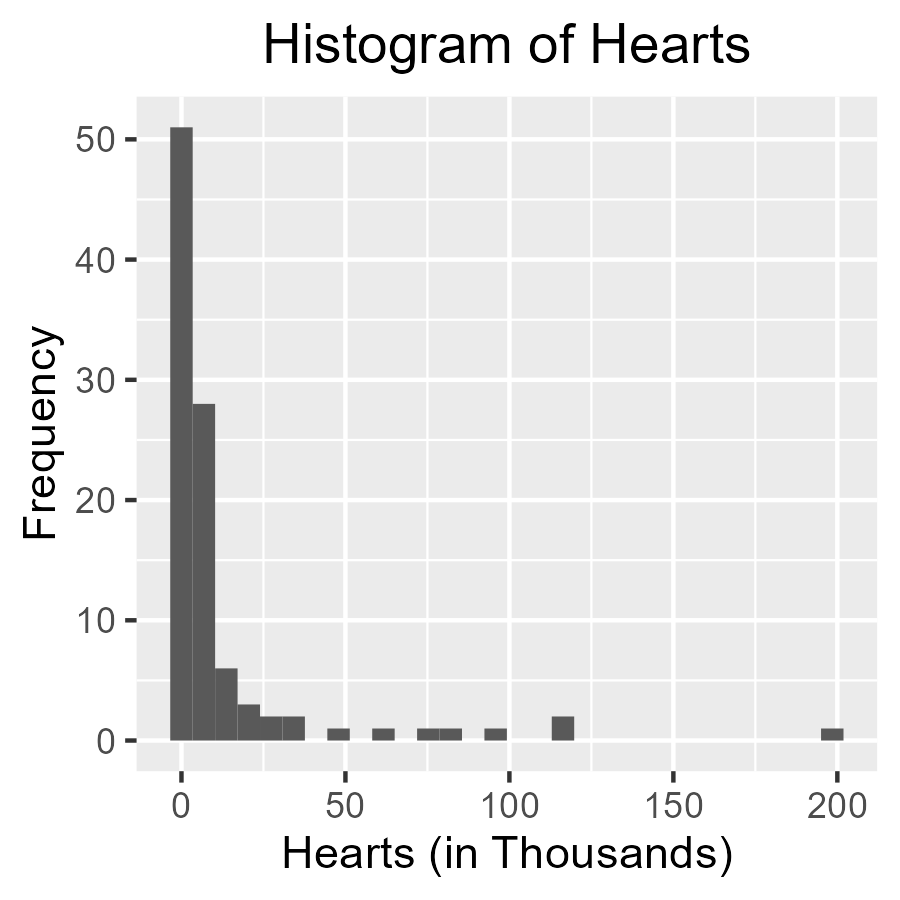

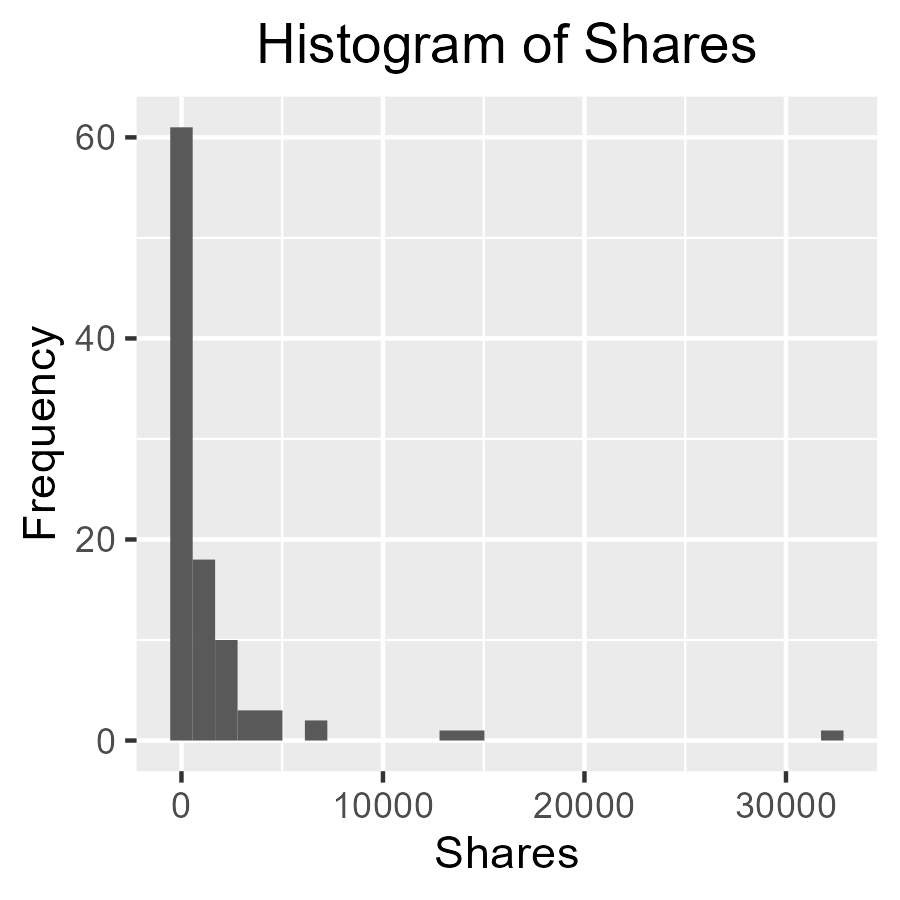

Supplement: Supplementary file 1 — Histograms [file mmc1.docx]
